# Multiple sclerosis: clinical features and MRI findings in Northern China

**DOI:** 10.1186/2047-783X-19-20

**Published:** 2014-04-15

**Authors:** Tianran Li, Hui Xiao, Shengjun Li, Xiangke Du, Jun Zhou

**Affiliations:** 1 Radiology Department, No. 1 Affiliated Hospital of Fuzhou General Hospital, 29 Xinquan Road, Putian, Fujian Province 351100, P.R. China; 2 Radiology Department, Fuzhou General Hospital, 156 Xi’erhuan Beilu, Fuzhou, Fujian Province 320025, P.R. China; 3 Neurology Department, Jinan General Hospital, 148 Jinyi Road, Jinan, Shandong Province 250001, P.R. China; 4 Radiology Department, Peking University People’s Hospital, 11 Xizhimen South Street, Beijing 100044, P.R. China

**Keywords:** Multiple sclerosis, Conventional multiple sclerosis, Optic-spinal cord multiple sclerosis, Magnetic resonance imaging

## Abstract

**Background:**

Reports in Asian populations suggest that ethnic and geographical differences may influence susceptibility to multiple sclerosis (MS) and its clinical behaviors. Here, we sought to retrospectively survey clinical characteristics and MRI data in Chinese subjects with MS.

**Methods:**

We conducted a retrospective analysis in 117 patients with MS. The patients were divided into subgroups with optic-spinal form of multiple sclerosis (OSMS; n = 42) and classical multiple sclerosis (CMS; n = 75). Clinical characteristics, MRI finding and expanded disability status scale (EDSS) score were compared between the two groups.

**Results:**

In 117 MS patients, 64.1% patients were classified as having CMS and 35.9% OSMS forms. White blood cell counts of OSMS patients were significantly higher than those of CMS patients (*P* <0.05). The longitudinal fusion lesions of spinal cord on MRI were statistically significant between groups (*P* <0.05). Spinal cord MRI showed that MS lesions were longer, and revealed spinal cord swelling in patients with CMS, but atrophy in patients with OSMS. The EDSS score at five years was significantly higher in patients with OSMS than in those with CMS (*P* <0.05). Relapse rates of patients with OSMS were also higher than those of patients with CMS (*P* <0.01) within one to three years.

**Conclusions:**

OSMS accounts for a higher proportion of MS populations in Northern China than in Western countries. MRI showed a longitudinally extensive spinal cord lesion in patients with OSMS and spinal cord swelling at onset.

## Background

Multiple sclerosis (MS) is an autoimmune disease of the central nervous system (CNS) characterized by the demyelination of white matter. It has been shown that racial and geographical differences may influence the susceptibility to MS as well as its manifestations, clinical characteristics, site of CNS involvement, pathogenesis and prognosis [1]. Multiple sclerosis is rare in Asian populations. Previous studies have shown that the incidence prevalence of MS in populations from Southern China (and Hong Kong, Taiwan) is low, (0.77 per 100,000 population) compared to that in Caucasians, with a high female to male ratio. These studies also showed that in Asia the site of CNS involvement was more commonly the optic nerve and spinal cord, and that the condition was characterized by relatively fast progression, with oligoclonal bands (OB) being found in the cerebrospinal fluid (CSF) of relatively few patients [2,3]. In MS populations from Southern China there is a high degree of spinal cord involvement (66%) [2], but comparatively little is known about the clinical characteristics of MS in Northern China.

The clinical course of MS can be classified as relapsing-remitting, primary-progressive, secondary-progressive, progressive-relapsing, benign and malignant MS forms [4]. There are thought to be two distinct phenotypes of relapsing-remitting MS in Asian populations. One is the conventional form of MS (CMS) that shares similar genetic and clinical features with the Western type of MS. The other is optic-spinal form of MS (OSMS), in which major neurological symptoms derive from optic neuritis [5]. However, this possibility remains a subject of controversy. Its clinical course is variable and unpredictable and its exact etiology is unknown. It is important to determine which MS phenotypes are present in Northern China and to identify any distinct clinical and magnetic resonance imaging (MRI) characteristics of MS in this population. We, therefore, retrospectively surveyed clinical data from MS patients presenting to the Chinese People’s Liberation Army General Hospital and Peking University People’s Hospital from 2002 to 2009. All patients included in the analysis were from the Northern Provinces of the Yangtze River.

## Methods

### Patients and classification

We conducted a retrospective analysis in 117 patients with MS (66 female, 51 male; average age 35.9 ± 11.8 years, range 15 to 71 years; average disease duration 3.9 ± 3.6 years, range 0.5 to 26 years). Each had a confirmed diagnosis of MS based on the revised McDonald 2005 criteria [6,7]. Patients with Sjogren’s syndrome, systemic lupus erythematosus, rheumatoid arthritis, vasculitis, malignancy, human T-cell leukemia virus type 1 or HIV were excluded from the analysis.

The patients were classified according to the course of the disease (relapse-remitting or, primary-progressive) [8] and the site of lesions. Relapsing-remitting MS was defined as neurological dysfunction that continued to deteriorate for at least 24 hours, and then became partially or completely stabilized, returning to baseline levels within one month. Primary-progressive (the equivalent of chronic progressive) MS was defined by progressive deterioration with no acute changes, over a period of at least six months [9]. Depending on the site of the disease, the patients were divided into optic-spinal or conventional forms of MS [5]. CNS lesions involving the cerebellum and brainstem were classified as CMS forms. Lesions mainly confined to the optic nerve and spinal cord lesions were classified as OSMS forms. Patients with small brain stem lesions were also classified as OSMS [10].

Neuromyelitis optica (NMO) is an uncommon neuroinflammatory syndrome that is distinct from MS. Differential diagnoses were discussed according to the multicenter analysis results and diagnostic criteria for neuromyelitis optica [11,12]. 1) Serologic antibody NMO-immunoglobulin G seropositive; 2) onset of NMO with strong female predilection; 3) NMO is recurrent longitudinally extensive transverse myelitis; 4) patients with NMO tend to initially present with optic neuritis.

#### Data analysis

Age, sex, initial clinical symptoms, physical signs, nervous system site and, if appropriate, the number of relapsing-remitting episodes were recorded. Results of CSF examination, including OB examination results, white blood cell counts, and total protein content were recorded. Evoked potential data were also included in the analysis. The expanded disability status scale (EDSS) was used to evaluate the degree of disability, based on the results of nervous system examinations [13].

#### Brain or spinal cord magnetic resonance imaging examination

Magnetic resonance sequences included conventional T1-weighted imaging, T2-weighted imaging, fluid-attenuated inversion recovery (FLAIR) sequence and diffusion-weighted imaging (DWI). The MRI diagnostic criteria for MS were those recommended in the European Magnetic Resonance Imaging in MS (MAGNIMS) proposal [14]. This required intravenous administration of a gadolinium-containing MR contrast agent, such as gadolinium-diethylenetriaminepentaacetic acid (Gd-DTPA), followed by dynamic contrast-enhanced MR imaging (DCE-MRI) of the brain or spinal cord. High-field MR images were obtained for all patients using a 1.5 or 3.0 Tesla MR Scanner.

### Statistical analysis

Statistical analysis was undertaken using the Statistical Package for Social Sciences (SPSS version 13.0) software (IBM Corp., Armonk, NY, USA). Initial symptoms, CFS fluid examination, MRI studies, evoked potential, the progress of disability (the EDSS), and relapse frequency were compared in the CMS and OSMS groups using chi-square or *t* tests, two-tailed. *P* values of less than 0.05 were declared to be significant.

## Results

### Multiple sclerosis classification and presenting symptoms

Of the 117 MS patients included in the study, 75 (64.1%) were classified as having CMS, and 42 (35.9%) were classified as having OSMS forms of MS. Six patients (5%) had primary progressive MS and 111 patients (95%) had relapsing-remitting MS.

The most common initial symptoms were physical signs of weakness (76.9%), sensory loss (66.7%), blurred vision (56.4%), sphincter dysfunction (43.6%) and paresthesia (see Table 1).

**Table 1:** Multiple sclerosis (MS) patients initial symptoms and physical signs

	**CMS (n = 75)**	**OSMS (n = 42)**	**MS (n = 117)**
Sex, F/M	38/37	28/14	66/51
Age, years	35.76 ± 12.23	36.12 ± 11.16	35.89 ± 11.81
**Symptoms, n (%)**			
Weakness	53 (70.7)	37 (88.1)	90 (76.9)
Sensory loss	42 (56.0)	36 (85.7)	78 (66.7)
Paresthesia	15 (20.0)	24 (57.1)	39 (33.3)
Blurred vision	34 (5.3)	32 (76.2)	39 (33.3)
Nystagmus	27 (36.0)	2 (4.7)	29 (24.8)
Ataxia	32 (42.7)	3 (7.1)	35 (29.9)
Sphincter symptoms	20 (26.7)	31 (73.8)	51 (43.6)
Diplopia	8 (10.7)	0	8 (6.8)
Dysarthria	12 (16.0)	0	12 (10.3)

CMS, conventional multiple sclerosis; OSMS, optic-spinal multiple sclerosis; MS, multiple sclerosis.

### Cerebrospinal fluid results

**Figure 1:**
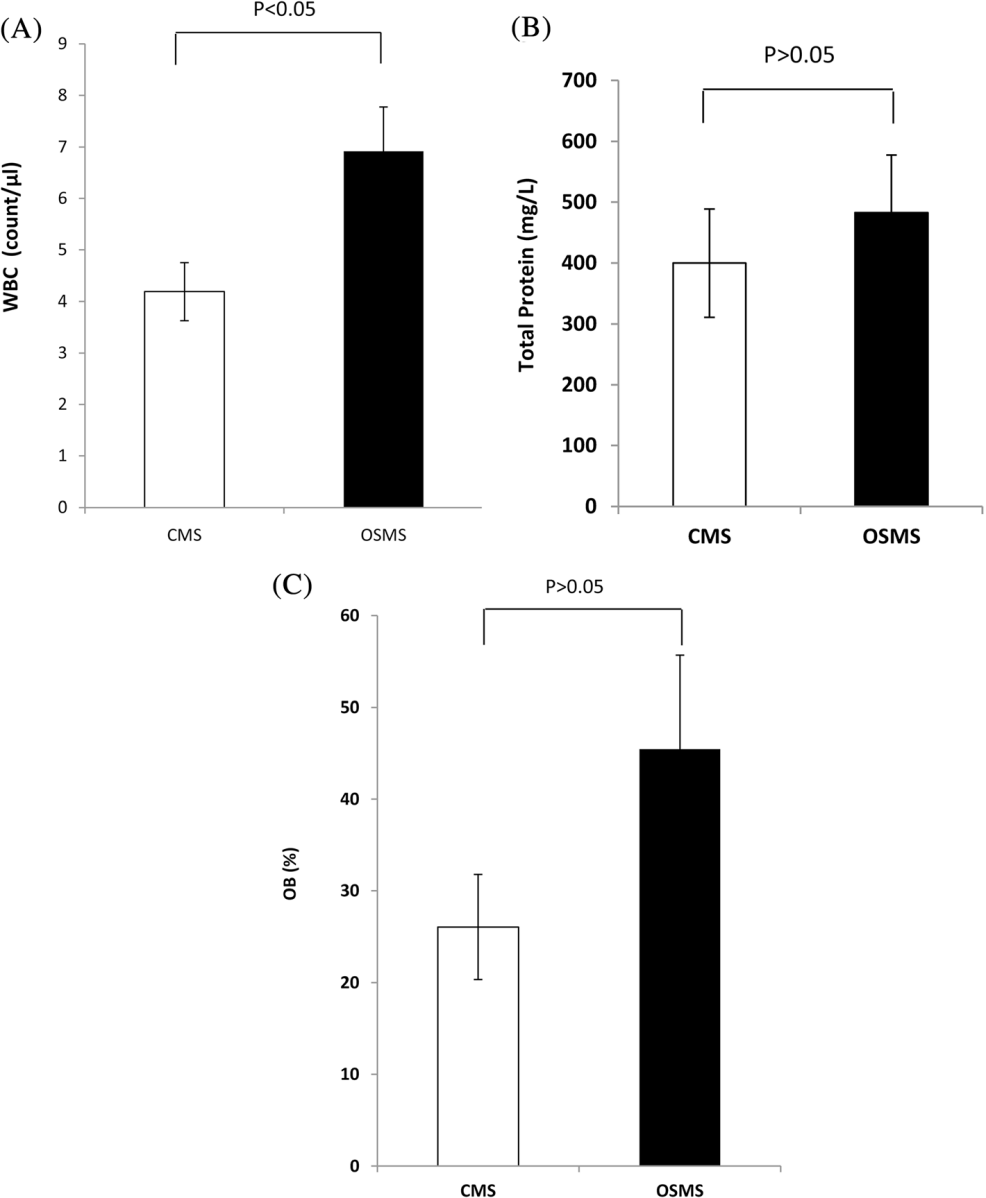
**Results of cerebrospinal fluid (CSF) examination, including white blood cell counts, total protein content, and oligoclonal band (OB). (B)** White blood cell count in CSF. The white blood cell counts were significantly higher in patients with optic-spinal multiple sclerosis (OSMS) patients than in those with conventional multiple sclerosis (CMS) (*P* <0.05). **(B)** Total protein content in CSF. The protein content of CSF was not statistically significant between groups. **(C)** OB in CSF. The OB of CSF was statistically significant between groups.

White blood cell counts were obtained in CSF of 83 patients (OSMS n = 24; CMS n = 59). White blood cell counts of OSMS patients were significantly higher than those of CMS patients (*P* = 0.03; Figure 1A).

The CSF protein results were available for 85 patients (OSMS n = 24; CMS n = 61), who had a lumbar puncture examination within one month of initial symptoms. The results indicated that the CSF protein content was higher in patients with OSMS patients than in those with CMS patients, but the difference was not statistically significant (*P* = 0.43; Figure 1B).

The CSF OB level was analyzed in 34 patients (OSMS n = 11; CMS n = 3). Five patients with OSMS were OB-positive (45.45%), and six patients with CMS were OB-positive (26.07%). There was no significant change of CSF OB-positive numbers between groups (*P* = 0.25; Figure 1C).

### Magnetic resonance imaging results

Thirty-four patients with OSMS and 29 with CMS underwent brain and spinal cord MRI examination within one week of presentation. Thirty-two of the 34 (94.1%) patients with OSMS and 14 of the 29 (48.2%) patients with CMS presented with longitudinal fusion of three or more vertebral segments and were classified as having extensive spinal cord lesions, and the lesion difference was statistically significant (*P* = 0.002).

All 63 patients with spinal cord lesions underwent Gd-DTPA-enhanced MRI evaluation. Seventeen patients with OSMS (50.0%) and 10 patients with CMS (34.5%) had lesions showing an enhancement pattern.

**Figure 2:**
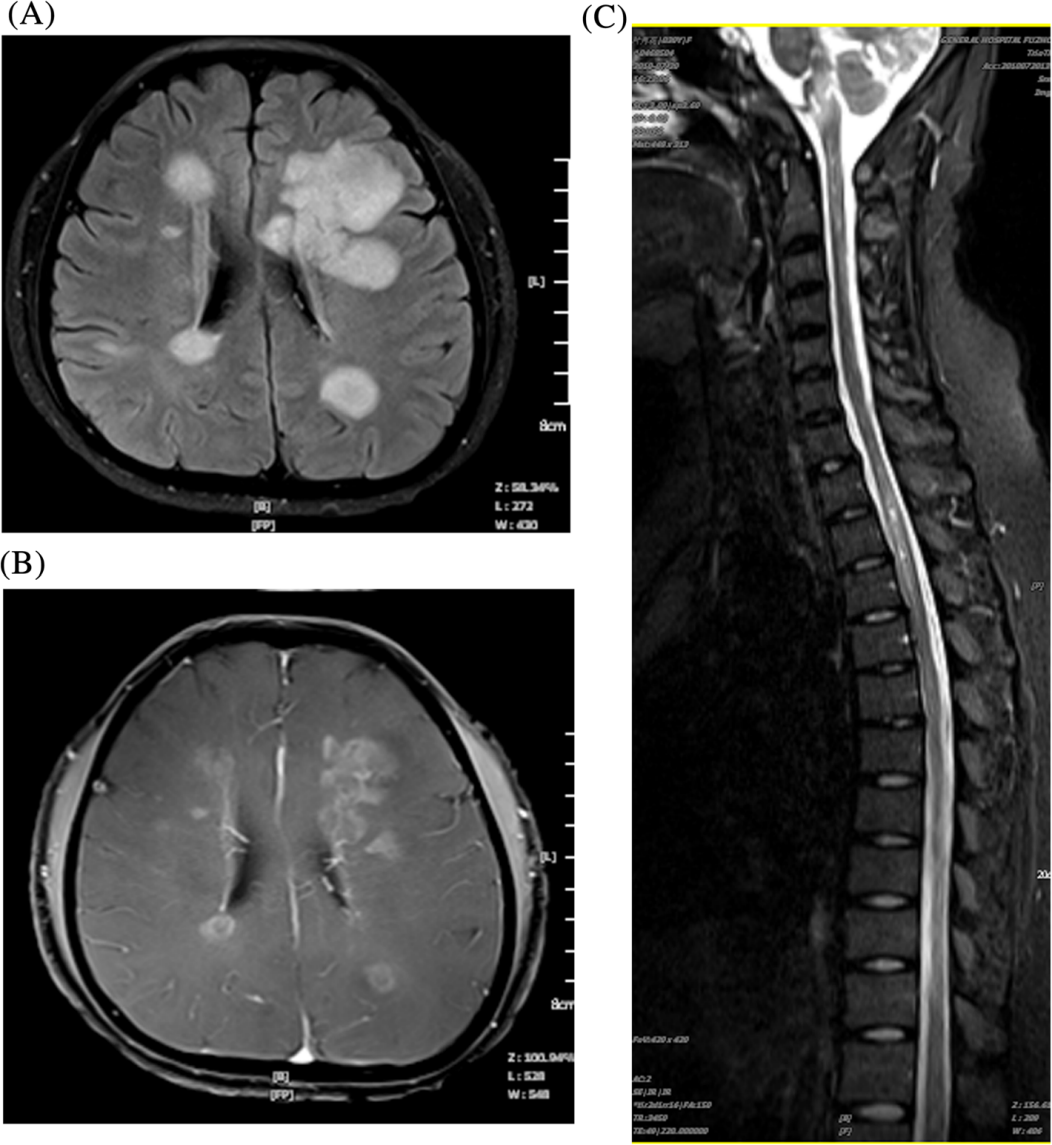
**Magnetic resonance imaging (MRI) scans from a 31-year-old male patient with conventional multiple sclerosis who presented with slow reaction times and memory loss for three days.** The patient had 97% small lymphocytes in the cerebrospinal fluid (CSF) and was oligoclonal band positive. **(A)** Periventricular lesions manifested hyperintense in fluid-attenuated inversion recovery (FLAIR) sequence. **(B)** Enhanced MRI T1-weighted image showed uniform nodular, circular lesions. **(C)** The lesions were also concentrated in the cervical and thoracic spinal cord, and were distributed along the long axis of the spinal cord and fat-suppressed T2-weighted image remained hyperintense.

MRI lesions in patients with CMS were concentrated in the periventricular, frontal, and parietal deep white matter, and manifested as round or confluent patch shadows. Periventricular lesion findings were a vertical distribution, indicating ‘right-angle’ demyelination. Routine MRI also detected a few cortical lesions in patients with CMS, predominantly in the gray matter-white matter junction (Figure 2A, B). Spinal cord MRI showed that CMS lesions were longer and showed increased cord swelling at onset. Lesions showed an abnormal signal, strip or prismatic, unclear, paralleling the long axis of the spinal cord (Figure 2C).

**Figure 3:**
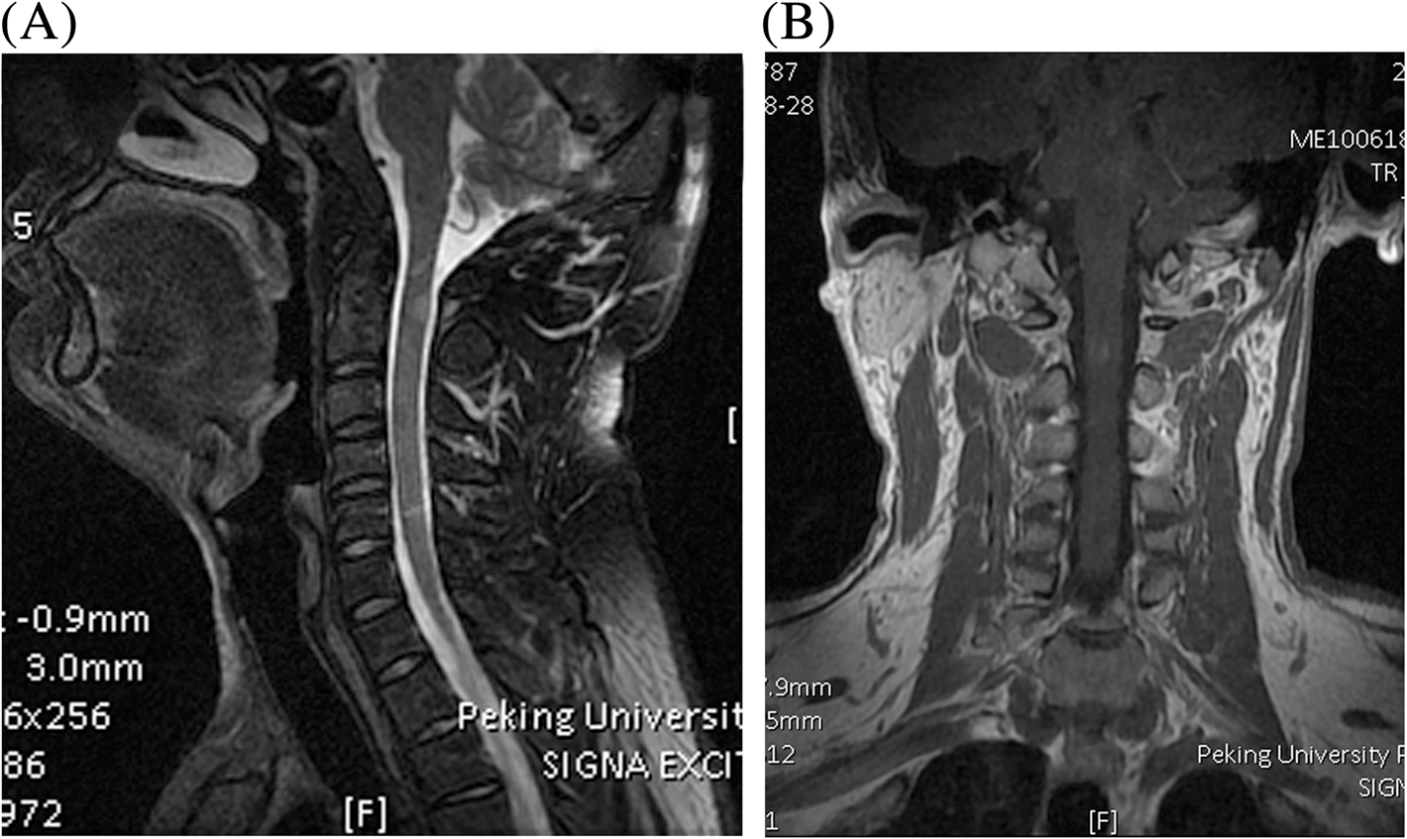
**Magnetic resonance imaging (MRI) scans from a 29-year-old male patient who presented with a five-week history of numbness and uncoordinated right upper limb activity for one week.** The patient had a nodular cervical spinal cord lesion. **(A)** T2-weighted imaging showed hyperintense and **(B)** enhanced MRI images identified uniform nodular lesions.

Spinal cord MRI showed that OSMS lesions were concentrated in the cervical and thoracic spinal cord, and were distributed along the long axis of the spinal cord. Spinal cord atrophy was a common feature in patients with OSMS. T1-weighted images were slightly hypointense, T2-weighted images were hyperintense showing a clear boundary, and the images remained hyperintense during FLAIR and DWI sequences (Figure 3A, B).

Periventricular lesions in patients with CMS are extended deeply into the white matter, while those in patients with OSMS are confined to periventricular areas and brainstem.

In CMS or OSMS, early lesions showed a uniform nodular structure on enhanced MRI. A few days to several weeks later, lesions appeared circular in shape on enhanced MRI and circular enhancement disappeared several weeks later. There was rapid changes in lesions after treatment.

### Evoked potential examination results

Nineteen patients with OSMS and 47 with CMS underwent somatosensory evoked potential (SEP) examination. Abnormal results were obtained in six patients (31.7%) with OSMS and in 20 patients (42.6%) with CMS patients.

Eighteen patients with OSMS and 42 with CMS had brainstem auditory evoked potential (BAEP) examinations. Two patients (11.1%) with OSMS patients and 12 patients (28.6%) with CMS had abnormal BAEP findings. Twenty-four patients with 24 OSMS and 50 with CMS patients had visual evoked potential (VEP) examinations. VEP findings were abnormal in 16 (56.0%) patients with OSMS and in 28 (59.5%) patients with CMS.

### Progression of disability

Five-year clinical data were available for 12 patients with OSMS and for 32 patients with CMS. EDSS scores calculated for these patients were significantly higher in patients with OSMS (2.42 ± 0.90) than in those with CMS (1.50 ± 0.88; *P* <0.01).

### Relapsing-remitting frequency

**Figure 4:**
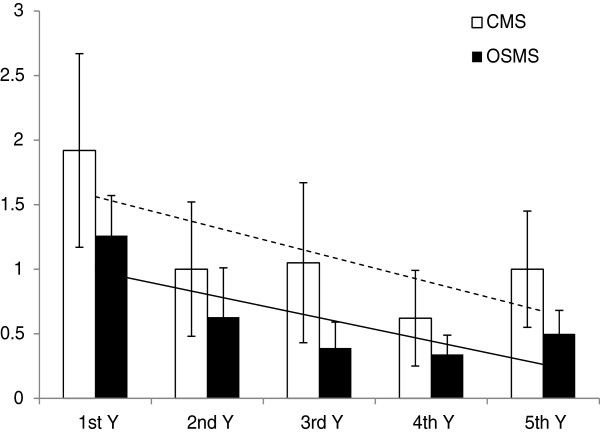
Five-year relapsing-remitting frequency per year in patients with OSMS (n = 12) and CMS (n = 32).

The annual relapsing-remitting frequency for the 44 patients with five-year clinical data is shown in Figure 4. In both groups there was a tendency for the frequency to decrease over the five-year observation period. However, at each time point the relapsing-remitting frequency was lower in the OSMS group than in the CMS group.

## Discussion

Multiple sclerosis (MS) is a common inflammatory autoimmune neurologic disorder and is the most frequent cause of nontraumatic neurologic disability in young and middle-aged adults. The clinical course of MS can be classified as relapsing-remitting, primary-progressive, secondary-progressive, progressive-relapsing, benign and malignant MS forms [4]. There are thought to be two distinct phenotypes of relapsing-remitting MS in Asian populations. Two distinct phenotypes of MS have been identified, which manifest as optic-spinal (OSMS) and conventional (CMS) forms of MS [5]. Research findings suggest that MS phenotypes are drastically altered by environmental factors, such as latitude and ‘Westernization’ [1]. In Asia, the proportion of MS patients with OSMS is the highest in the world, and is much higher than in the Caucasian population [15]. Published data also suggest that OSMS is more common in female patients that CMS, and that the degree of disability progresses faster, with more relapses, especially in the first and third years. However, even within Asia, there are inconsistent incidence reports for CMS and OSMS. In a series of 1,493 Japanese patients studied in 2009, 57.7% were classified as having CMS and 16.5% were as having OSMS in [16]. However, in 2006 it was estimated that OSMS accounted for 56% of Taiwanese patients with MS [17].

In our population of patients from Northern China, 35.9% had OSMS and 64.1% had CMS. The patients with OSMS had severe spinal cord lesions and a few had brain lesions. Only 5% of patients in our study had primary-progressive MS, which is in accordance with previously published data [17]. This also contrasts with Western Caucasian populations, where more than 30% of MS patients have primary-progressive disease [18].

In both OSMS and CMS patients, the most common clinical symptom at onset was limb weakness. In the OSMS group, there were no cases of diplopia or dysarthria, and only two patients had nystagmus. However, significantly more patients with OSMS than CMS had sphincter disorders as a presenting symptom.

White blood cell count in the CFS of the OSMS group was higher than that of the CMS group. It indicated that there has been acute inflammatory process and it is a more urgent task to control inflammation in OSMS patients. It has previously been reported that CSF protein levels are significantly higher in patients with OSMS than in those with CMS [16]. In our patients, CSF protein levels were numerically higher in the OSMS group than in the CMS group but the difference was not statistically significant. This finding may be related to the fact that some patients in our study did not have a lumbar puncture performed within three days of the onset of presenting symptoms. OBs are also important disease indicators in MS patients with both OSMS and CMS. In our study, OB testing was not as seen in Caucasian populations. Indeed, some studies have speculated that the differences in occurrence of OB between Eastern and Western populations may be the consequence of a different immune response of patients with different genetic backgrounds [19-21].

MRI has played a unique role in the diagnosis and management of MS. In recent years, there have been considerable changes in the diagnostic criteria for MS as MRI-based studies have enabled earlier and more accurate diagnosis of the disease [22]. Total brain lesion volume and spatial distribution of lesions across the brain vary widely between patients. Anterior periventricular linear lesions and the absence of ovoid lesions are characteristic of OSMS. In addition, longitudinally extensive spinal cord lesions affecting three or more vertebral segments are significantly more common in patients with OSMS than in those with CMS. However, in our study some of the CMS patients also had longitudinally extensive spinal cord lesions. These findings suggest that in Asian populations, longitudinally extensive spinal cord lesions are heterogeneous between OSMS and CMS patients.

Noninvasive MRI evaluation of the blood–brain barrier (BBB) is important for monitoring disease progression and evaluating therapeutic efficacy. MRI enhancement of MS lesions indicates breakdown of the BBB by active lesions, which is a feature of MS. In our study, we found only a few cortical lesions located in the gray matter-white matter junction of patients with CMS. Other studies have reported that the presence of cortical lesions confers physical disability and cognitive impairment [23].

Evoked potentials assist early diagnosis of MS by detecting subclinical lesions. In our study, results from high sensitivity VEP tests, and from low sensitivity, was brainstem auditory evoked potentials were consistent with patients’ initial symptoms, and indicated minimal brain stem involvement in most patients.

The most significant clinical feature of MS is that of relapse. We found that relapse frequency in the first and third year after clinical onset was significantly higher in patients with OSMS than in those with CMS. The relapse frequency was also numerically higher than in CMS patients in the second, fourth and fifth years after clinical onset. The degree of disability progressed faster and was more likely to result in relapse, emphasizing the importance of early diagnosis and intervention.

## Conclusions

In summary, we have shown that OSMS accounts for a higher proportion of MS patients in Northern China than in Western countries. In both OSMS and CMS patients, the most common clinical symptom was limb weakness at onset. White blood cell count in the CSF of OSMS patients was high, which may indicate that there had been an acute inflammatory process. MRI showed a longitudinally extensive spinal cord lesion in patients with OSMS.

## Consent

Written informed consent was obtained from the patients for the publication of this report and any accompanying images.

## Abbreviations

BAEP: brainstem auditory evoked potential; BBB: blood–brain barrier; CMS: conventional multiple sclerosis; CNS: central nervous system; CSF: cerebrospinal fluid; DCE-MRI: dynamic contrast-enhanced magnetic resonance imaging; DWI: diffusion-weighted imaging; EDSS: Expanded Disability Status Scale; FLAIR: fluid-attenuated inversion recovery; Gd-DTPA: gadolinium-diethylenetriaminepentaacetic acid; MRI: magnetic resonance imaging; MS: multiple sclerosis; NMO: neuromyelitis optica; OB: oligoclonal band; OSMS: optic-spinal form of multiple sclerosis; SEP: somatosensory evoked potential; VEP: visual evoked potential.

## Competing interests

The authors declare that they have no competing interests.

## Authors’ contributions

TRL designed the clinical trial, acquired and analyzed the data, and drafted the manuscript. HX designed the clinical trial. SJL designed the clinical trial, collected patient data, and analyzed the data. XKD acquired the data and drafted the manuscript. All authors read and approved the final manuscript. JZ: contributed to the conception and design, administrative support.
